# Effects of Cranberry Juice Supplementation on Cardiovascular Disease Risk Factors in Adults with Elevated Blood Pressure: A Randomized Controlled Trial

**DOI:** 10.3390/nu13082618

**Published:** 2021-07-29

**Authors:** Chesney K. Richter, Ann C. Skulas-Ray, Trent L. Gaugler, Stacey Meily, Kristina S. Petersen, Penny M. Kris-Etherton

**Affiliations:** 1 Department of Nutritional Sciences, University of Arizona, Tucson, AZ 85716, USA; richterck@email.arizona.edu (C.K.R.); skulasray@email.arizona.edu (A.C.S.-R.); 2 Department of Mathematics, Lafayette College, Easton, PA 18042, USA; gauglert@lafayette.edu; 3 Department of Nutritional Sciences, Pennsylvania State University, University Park, PA 16802, USA; sas117@psu.edu (S.M.); Kristina.Petersen@ttu.edu (K.S.P.); 4 Department of Nutritional Sciences, Texas Tech University, Lubbock, TX 79409, USA

**Keywords:** lipids, LDL-C, blood pressure, arterial stiffness, inflammatory markers

## Abstract

Emerging cardiovascular disease (CVD) risk factors, including central vascular function and HDL efflux, may be modifiable with food-based interventions such as cranberry juice. A randomized, placebo-controlled, crossover trial was conducted in middle-aged adults with overweight/obesity (*n* = 40; mean BMI: 28.7 ± 0.8 kg/m^2^; mean age: 47 ± 2 years) and elevated brachial blood pressure (mean systolic/diastolic BP: 124 ± 2/81 ± 1 mm Hg). Study participants consumed 500 mL/d of cranberry juice (~16 fl oz; 27% cranberry juice) or a matched placebo juice in a randomized order (8-week supplementation periods; 8-week compliance break), with blood samples and vascular measurements obtained at study entry and following each supplementation period. There was no significant treatment effect of cranberry juice supplementation on the primary endpoint of central systolic blood pressure or central or brachial diastolic pressure. Cranberry juice significantly reduced 24-h diastolic ambulatory BP by ~2 mm Hg compared to the placebo (*p* = 0.05) during daytime hours. Cranberry juice supplementation did not alter LDL-C but significantly changed the composition of the lipoprotein profile compared to the placebo, increasing the concentration of large LDL-C particles (+29.5 vs. −6.7 nmol/L; *p* = 0.02) and LDL size (+0.073 vs. −0.068 nm; *p* = 0.001). There was no effect of treatment on ex vivo HDL efflux in the total population, but exploratory subgroup analyses identified an interaction between BMI and global HDL efflux (*p* = 0.02), with greater effect of cranberry juice in participants who were overweight. Exploratory analyses indicate that baseline C-reactive protein (CRP) values may moderate treatment effects. In this population of adults with elevated blood pressure, cranberry juice supplementation had no significant effect on central systolic blood pressure but did have modest effects on 24-h diastolic ambulatory BP and the lipoprotein profile. Future studies are needed to verify these findings and the results of our exploratory analyses related to baseline health moderators.

## 1. Introduction

Cardiovascular disease (CVD) and related co-morbidities remain the leading cause of death worldwide [1,2]. Current dietary guidelines for reducing CVD risk recommend dietary patterns that emphasize bioactive-rich, plant-based foods [3,4]. Cranberries have a unique phytochemical profile and are a particularly good source of proanthocyanins, anthocyanins, flavonoids, and phenolic acids [5,6]. In vitro studies have shown that cranberry polyphenols exert strong anti-inflammatory and antioxidant properties [5,7]. However, this does not always translate directly to significant clinical effects, and additional research is needed to identify which CVD risk factors may be modifiable with cranberry juice supplementation.

Previous studies of cranberry supplementation have placed little emphasis on blood pressure and vascular function outcomes. A recent meta-analysis of cranberry supplementation studies found no effect on diastolic blood pressure, but systolic blood pressure was significantly lowered (−4 mm Hg), particularly in participants over 50 years old [8]. Beyond brachial blood pressure, central blood pressure and indices of arterial stiffness are more accurate predictors of cardiovascular events and disease severity [9,10]. To our knowledge, only two previous cranberry supplementation studies have investigated these endpoints. Low-calorie cranberry juice had no effect on augmentation index (AI) in overweight men compared to the control [11], while double-strength cranberry juice (54% juice) significantly reduced pulse wave velocity (PWV) in patients with stable coronary artery disease [12].

Cranberry bioactives may also be beneficial for atherogenic lipoproteins. For instance, cranberry juice has been shown to significantly increase high-density lipoprotein-cholesterol (HDL-C) in adults with abdominal obesity [13], reduce triglycerides in healthy adults [14], and reduce apolipoprotein B while increasing apolipoprotein A-I in patients with type 2 diabetes [15]; however, these effects were not observed in other studies of cranberry juice [16,17,18] and cranberry extracts [19]. In a recent meta-analysis of studies using a variety of different types of cranberry supplements (*n* = 10), there was a significant increase in HDL-C, particularly for participants less than 50 years of age, but there was no effect on total cholesterol, LDL-C, or triglycerides [8]. HDL-C efflux capacity may also be a more accurate measure of CVD risk than HDL-C concentration [20] and warrants investigation related to cranberry supplementation.

The present study was designed to investigate whether daily consumption of cranberry juice (16 fl oz per day) for 8–12 weeks could improve specified CVD risk factors in adults with elevated blood pressure. We hypothesized that cranberry juice would significantly improve vascular function, reduce atherogenic lipoproteins, and improve HDL function.

## 2. Materials and Methods

### 2.1. Study Population

Men and women who were 30–65 years of age, had elevated resting blood pressure (systolic blood pressure ≥120 mm Hg and/or diastolic blood pressure ≥80 mm Hg and <160/100 mm Hg), had a BMI of ≥18 and ≤39 kg/m^2^, and otherwise free of any serious illness were recruited for the study. Other inclusion criteria included fasting TG (<350 mg/dL) and total cholesterol (<284 mg/dL for women and <273 mg/dL for men). Exclusion criteria included smoking and/or use of other tobacco products; a history of diabetes, autoimmune disorders, and heart, liver, kidney, or uncontrolled thyroid disease; pregnancy, lactation, or a desire to become pregnant during the study; and chronic use of non-steroidal anti-inflammatories or medications/supplements for elevated lipids, blood pressure, or glucose.

### 2.2. Participant Recruitment

Participants were recruited and consented for the study from December 2015 to October 2018. Recruitment activities included fliers in the community, campus e-mail lists, local newspaper advertisements, and a university research website. Potential subjects emailed or called to indicate interest in participating and were then given additional information about the study. If interested, they were asked a series of medical history and lifestyle questions to screen for eligibility. After written informed consent was obtained, a urine pregnancy test was performed for women of child-bearing potential, and blood pressure was measured according to JNC 7 Guidelines [21]. Briefly, after a 5-min seated rest, three readings were taken by nurses in a controlled environment using a calibrated mercury sphygmomanometer. The mean of the last 2 readings was used to determine eligibility. If an individual’s blood pressure met the study inclusion criteria, body weight and height were measured (without shoes and in light clothing) to calculate BMI. A blood sample was then drawn for a complete blood count and standard chemistry profile (lipid panel, glucose, liver, and kidney function) to rule out the presence of illness (autoimmune disease, cancer, and immunodeficiency). A balanced randomization scheme with a block size of four was developed in advance (by Dr. Ann Skulas-Ray) using an online randomization generator, and subjects were assigned to a treatment sequence at enrollment (by Danette Teeter and Stacey Meily). Sample size was determined based on a power calculation, with central systolic blood pressure as the primary outcome. Based on variability of central systolic blood pressure in our previous work [22], it was estimated that 34 participants would provide 80% power to detect a 4.5 mm Hg change with a significance level of 0.05. This study was conducted according to the guidelines issued in the Declaration of Helsinki, and all procedures involving human subjects were approved by the Institutional Review Board of the Pennsylvania State University. Written informed consent was obtained from all participants. The study is registered at clinicaltrials.gov as NCT02556749.

### 2.3. Study Design and Intervention

This was a randomized, placebo-controlled, 2-period crossover study with 8-week treatment periods separated by an approximate 8-week compliance break. Treatment periods were extended by up to 4 weeks (12 weeks total) in the case of illness, injury, or scheduling difficulties. During the treatment periods, participants received 500 mL/d (~16 fl oz) of cranberry juice (~70 kcal; 27% cranberry juice) or 500 mL/d (~16 fl oz) of a placebo juice (matched for color, calorie, and carbohydrates), in random order. Additional information regarding the nutrient profile, bioactive content, and ingredients of the study juices can be found in Tables S1 and S2. The placebo beverage was formulated to match appearance, aroma, and taste of the cranberry juice and to have a similar amount of total calories, total carbohydrates, and total organic acids. Treatments consisted of individually packaged juice provided in identical bottles during both periods. Treatments were matched to coded alphanumeric identifiers so that the investigators and participants were blinded to treatment assignment. The juices were matched for taste and appearance to maintain the blinding of participants and investigators to treatment sequence. All juices were formulated and provided by Ocean Spray (Middleborough, MA, USA).

Participants were instructed to consume two bottles of juice per day during each treatment period, either all at once or divided throughout the day (e.g., 8 fl oz with breakfast and 8 fl oz with dinner). They were provided with two weeks’ worth of juice at a time and were asked to incorporate the study juices into their habitual diet, maintaining their normal dietary intake and physical activity. In addition, participants were instructed to avoid consuming any other food or drinks that contained cranberry or cranberry juice for the duration of the study. Compliance with the study interventions was assessed via self-report. Participants completed a daily consumption log to track their consumption of the study juice each day. Participants reported to the Clinical Research Center on a biweekly basis. At this time, a member of the research team who was not involved in the statistical analyses provided participants with additional study juice and reviewed their body weight and juice consumption logs.

Blood sampling and vascular testing were performed at baseline and following each 8-week supplementation period. All study procedures were conducted at the Pennsylvania State University CRC according to standardized protocols. During the 48 h prior to testing visits, participants were instructed to avoid alcohol; refrain from taking non-approved medications and supplements, including pain relievers, vitamins, or minerals; and limit their intake of coffee and tea to no more than 1 standard beverage per day. Strenuous exercise was avoided for 12 h prior to testing. Testing visits were conducted following an overnight fast (no food or drink other than water for 12 h). Endpoint measurements were conducted on two consecutive days. On one day, vascular function testing was performed prior to blood sampling. On the other day of testing, only a fasting blood draw was performed. Pre-menopausal women were scheduled for vascular testing within the first 7 days of starting their menstrual period in order to minimize hormonal effects on vascular endpoints. Ambulatory blood pressure was also measured at baseline and the end of each supplementation period, with the device worn for a 24-h period on each occasion. Typically, participants were fitted with the device on the first day of testing and returned it on the following testing day. All study procedures were completed in May 2019. Participants received monetary compensation of $200 for completion of the study.

### 2.4. Vascular Function Measures

Central blood pressure and arterial stiffness indices were assessed using the SphygmoCor XCEL System pulse waveform analysis (AtCor Medical, Sydney, Australia). All measurements were performed in a temperature-controlled, quiet, dimly lit room.

### 2.5. Pulse Wave Analysis (PWA): Central (Aortic) Blood Pressure and Augmentation Index (AI)

Following a 5-min seated rest, central pressures and wave reflection characteristics (i.e., augmentation pressure (AP) and AI) were derived from brachial pressure waveforms using a generalized transfer function that is considered to be substantially equivalent to generalized transfer functions for radial tonometry validated against an indwelling catheter [23,24,25]. At each visit, three PWA measurements were taken, following JNC 7 Blood Pressure Guidelines [21], with 1 min between each reading. The last two PWA results were averaged and used for analysis. The AI was standardized to a heart rate of 75 beats per minute (AI@75) to correct for the independent inverse effect of heart rate on augmentation of the pulse wave form [26].

### 2.6. Pulse Wave Velocity (PWV)

Aortic stiffness was assessed by carotid-femoral pulse wave velocity (PWV). Carotid and femoral arterial pressure waveforms were measured simultaneously via an applanation tonometry sensor manually held in place above the right common carotid artery and a blood pressure cuff placed on the right femoral artery. Distance measurements were taken from the sternal notch to the carotid artery, from the sternal notch to the top of the femoral cuff and from the femoral artery to the top of the femoral cuff. Based on these measurements, the SphygmoCor XCEL System automatically calculates the distance traveled by the pulse wave from the carotid artery to the femoral artery. Transit time between the carotid and femoral pressure waves is determined by the SphygmoCor System using the foot-to-foot method [27]. PWV is then calculated as distance over transit time. At each visit, three PWV measurements were obtained in the supine position, with 1 min between readings. The last two PWV results were averaged for analysis.

### 2.7. Ambulatory Blood Pressure Monitoring

Participants wore an ambulatory blood pressure monitor for a 24-h period at baseline and the end of each treatment period. Following baseline day 1 testing and during the last week of each treatment period, the monitor (Mortara Instrument Inc., Milwaukee, WI) was fitted by study personnel on the non-dominant arm and programmed to automatically capture a reading every 20 min during the day and every 30 min overnight (10 p.m. to 6 a.m.). The mean number of successful readings obtained in a 24-h period was 50 ± 10, with 36 ± 8 awake readings and 13 ± 5 asleep readings.

### 2.8. Blood Sample Collection and Assay Methods

Blood drawn into anticoagulant-coated tubes containing lithium heparin or EDTA was immediately centrifuged for 15 min at 1500× *g*. Blood drawn into serum separator tubes was allowed to clot for 30 min prior to centrifugation. Total cholesterol and triglycerides (TG) were measured by enzymatic procedures (Quest Diagnostics, Pittsburgh, PA, USA; CV < 2% for both). HDL-C was estimated according to the modified heparin-manganese procedure (Quest Diagnostics; CV < 2%). LDL-C was measured directly via enzymatic analysis. Glucose was determined by Spectrophotometry procedures (Quest Diagnostics). Insulin was measured by radioimmunoassay using ^125^I-labeled human insulin and a human insulin antiserum (Quest Diagnostics). Serum high-sensitivity C-reactive protein (CRP) was measured by latex-enhanced immunonephelometry (Quest Diagnostics; assay CV < 8%). For other endpoints, aliquots of serum and plasma were immediately stored at −80 °C for batch analysis. Plasma lipoprotein particle number and size were assessed by a proton magnetic resonance spectroscopy assay (NMR LipoProfile III; LipoScience, Raleigh, NC, USA), which measures the particle concentrations of lipoprotein subclasses and mean particle size of lipoproteins. Plasma isoprostane concentrations were measured by the Vanderbilt University Eicosanoid Core Laboratory using gas chromatography/negative-ion chemical ionization mass spectrometry (GC/NICI-MS), as described previously [28].

### 2.9. Cholesterol Efflux

Serum HDL (apoB-depleted serum) was prepared from individual serum samples by precipitation of apoB-containing lipoproteins using polyethylene glycol (PEG). Briefly, for each serum sample, 100 parts serum is mixed with 40 parts PEG (20%, *v*/*v*, in glycine buffer, pH 7.4). The mixture is incubated at room temperature for 20 min and then centrifuged at 10,000 rpm for 30 min at 4 °C. The supernatant containing serum HDL is collected and used for analysis of cholesterol efflux capacity. Cholesterol efflux capacities of serum HDL samples were determined as described in detail elsewhere [29,30]. In brief, global and ABCA1-mediated cholesterol efflux were measured using J774 mouse macrophage cells in the presence and/or absence of cAMP. For all assays, cells were pre-incubated with [^3^H]-cholesterol and ACAT inhibitor Sandoz 58-035 (but not preloaded with mass cholesterol) overnight. Cells were then incubated overnight in 0.2% BSA with or without cpt-cAMP. After washing, the cells were incubated for 4 h with the serum HDL samples (apoB-depleted serum) added at 2.8% (*v*/*v*). [^3^H]-cholesterol released to serum after incubation with cells for 4 h was measured by liquid scintillation counting. Cholesterol efflux is expressed as the radiolabel released as a percentage of [^3^H]-cholesterol within cells before addition of serum. All efflux values were corrected by subtracting the small amount of radioactive cholesterol released from cells incubated with serum-free medium. The global cholesterol efflux from J774 cells treated with cAMP includes cholesterol efflux mediated by ABCA1, SR-BI, ABCG1, passive diffusion, or other still unknown carriers [30]. ABCA1-dependent efflux from J774 cells was determined as the difference in efflux from cAMP-treated and untreated cells. Cholesterol efflux from untreated cells is mediated by SR-BI, ABCG1, passive diffusion, or other still unknown carriers.

### 2.10. Statistical Analyses

All statistical analyses were performed using SAS (version 9.4; SAS Institute). Only participants who completed both supplementation periods were included in analyses. Participants with CRP values ≥ 10 mg/L (*n* = 4) were excluded from analyses of CRP, as these values are indicative of acute inflammation from an isolated physical injury and/or infection [31]. Differences between male and female participants at baseline were assessed via independent two-sample *t*-test (PROC TTEST). Change scores for end-of-treatment values were calculated by subtracting study-entry baseline values from each post-supplementation measure. For end-of-treatment values, the mixed models procedure (PROC MIXED) in SAS was used to test the effects of treatment, period, and treatment by period interactions for each outcome. Outcome variables were assessed for normality (PROC UNIVARIATE). Baseline values were included as covariates for each endpoint. Outcomes were modeled as repeated measures, with subject as a random effect and with unstructured variance for treatment/period. Treatment by period interactions were included in order to test for potential carry-over effects between intervention periods. If significant, the treatment by period interaction was retained in the model, and lower order effects of treatment and period were not interpreted. When period and treatment by period interactions were nonsignificant, they were removed from the model. When period effects were significant, they were retained in the final model of treatment effects. Values that were measured in duplicate on separate days (i.e., body weight and lipids) were averaged prior to analysis. Subgroup analyses were conducted for measures of HDL efflux to investigate the influence of BMI on treatment effects. Participants were categorized as normal weight (BMI: 18.5–24.9 kg/m^2^), overweight (BMI: 25–29.9 kg/m^2^), or obese (BMI: ≥ 30 kg/m^2^). An exploratory analysis was also conducted to investigate the effect of baseline CRP on treatment effects. Participants were split into high and low CRP categories based on the median baseline CRP value of 1.3 mg/L. Means are reported as least-squares means ± SEM (standard error of the mean). For all tests, α was set at 0.05.

## 3. Results

**Figure 1:**
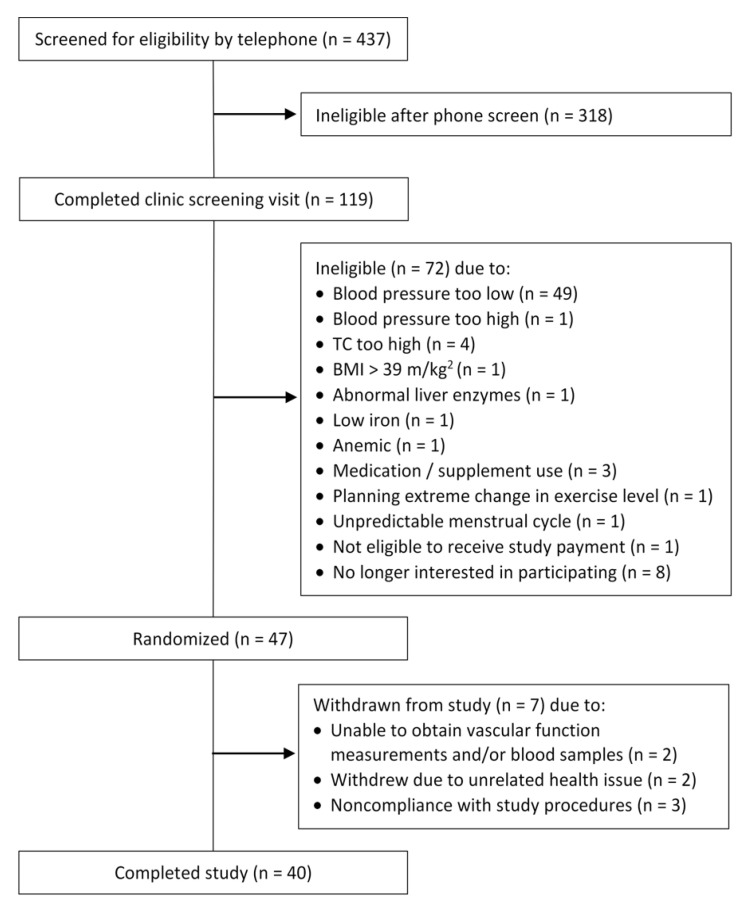
Schematic of participant recruitment and exclusion.

A schematic of participant recruitment for the study is provided in Figure 1. Of the 119 individuals who were screened, 47 met eligibility criteria and were enrolled in the study. Seven participants withdrew during the study due to unrelated health concerns (*n* = 2), inability to obtain study measurements (*n* = 2), and noncompliance with study protocol (*n* = 3). Thus, data are reported for 40 participants. Based on completion of self-reported daily consumption logs, compliance with the study protocol was 90%.

The screening characteristics of participants who completed the study are presented in Table 1. At baseline, there was a significant difference between female (*n* = 15) and male (*n* = 25) participants for select blood pressure and lipid/lipoprotein measurements. Female participants were significantly older (53 ± 11 years vs. 43.5 ± 11 years; *p* = 0.01) and had higher HDL-C (56 ± 11 mg/dL vs. 44 ± 10 mg/dL; *p* = 0.002), central systolic blood pressure (119 ± 12 mm Hg vs. 112 ± 7 mm Hg; *p* = 0.05), pulse pressure (39 ± 7.5 mm Hg vs. 30 ± 4 mm Hg; *p* = 0.0004), augmentation pressure (13.3 ± 5.2 mm Hg vs. 6.1 ± 3.1 mm Hg; *p* < 0.0001), and augmentation index (30.8 ± 23.3 % vs. 16.5 ± 12.2 %; *p* = 0.0005) values. Men had significantly higher triglycerides (122 ± 48 mg/dL vs. 90 ± 17 mg/dL; *p* = 0.005). Significant treatment by sex interactions were found for total cholesterol (interaction *p* = 0.04), medium HDL particles (interaction *p* = 0.007), and small HDL particles (interaction *p* = 0.02) (Figure S1). There was no significant effect of supplementation on weight. Baseline values according to treatment randomization order are also presented in Table 1. There were no significant differences according to treatment order for any variables (*p* > 0.05).

### 3.1. Effect of Cranberry Juice on Measures of Vascular Function

**Figure 2:**
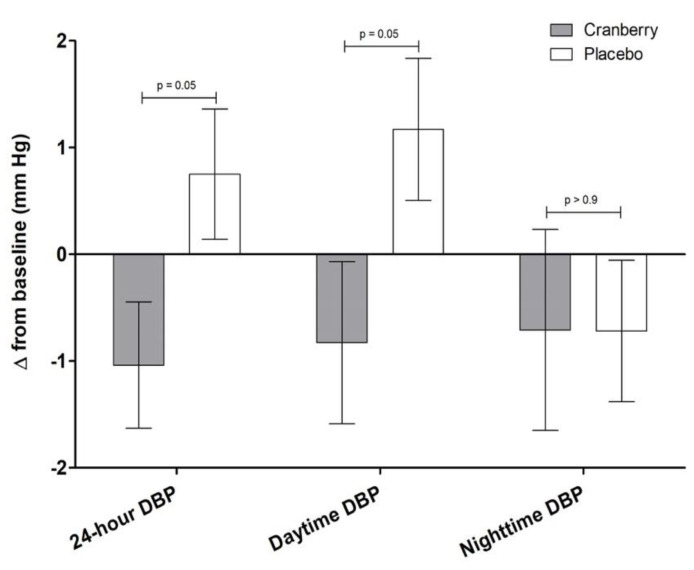
Change in ambulatory diastolic blood pressure following supplementation (*n* = 40). Change scores were calculated by subtracting study-entry baseline values from each post-supplementation measure. Values are means ± SEM (*n* = 40; 25 M, 15 F) and were compared using the MIXED models procedure (SAS version 9.4; SAS Institute Inc., Cary, NC, USA) to test the effects of treatment, period, and treatment by period interactions. Baseline values were included as covariates for each endpoint. *p*-values represent significant main effects of treatment.

There were no significant main effects of treatment for the primary endpoint of central systolic blood pressure, central or brachial diastolic blood pressure, nor for any other measures of brachial or central blood pressure, augmentation index, and pulse wave velocity (Table 2). Cranberry supplementation significantly reduced ambulatory diastolic blood pressure compared to the placebo (mean difference = −1.79 ± 0.9 mm Hg; *p* = 0.05), with the change occurring during awake, daytime hours (mean difference = 2.0 ± 1.0 mm Hg; *p* = 0.05) and not during the nighttime period (*p* > 0.9) (Figure 2). Cranberry juice did not alter any other ambulatory blood pressure values.

### 3.2. Effects of Cranberry Juice on Atherogenic Lipoproteins and Other Blood Markers of CVD Risk

**Figure 3:**
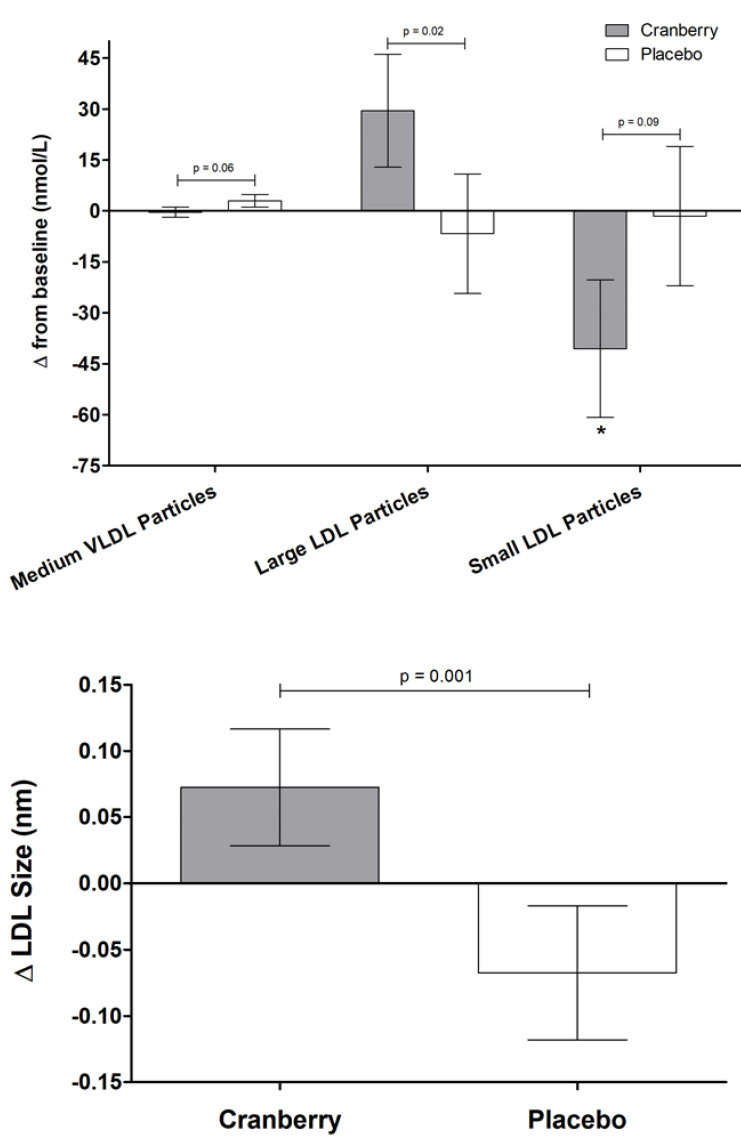
Changes in lipoprotein sub-fractions and characteristics following supplementation. Change scores were calculated by subtracting study-entry baseline values from each post-supplementation measure. Values are means ± SEM (*n* = 40; 25 M, 15 F) and were compared using the MIXED models procedure (SAS version 9.4; SAS Institute Inc., Cary, NC, USA) to test the effects of treatment, period, and treatment by period interactions. Baseline values were included as covariates for each endpoint. *p*-values represent treatment effects, and * indicates a significant change from baseline (*p* = 0.05).

**Figure 4:**
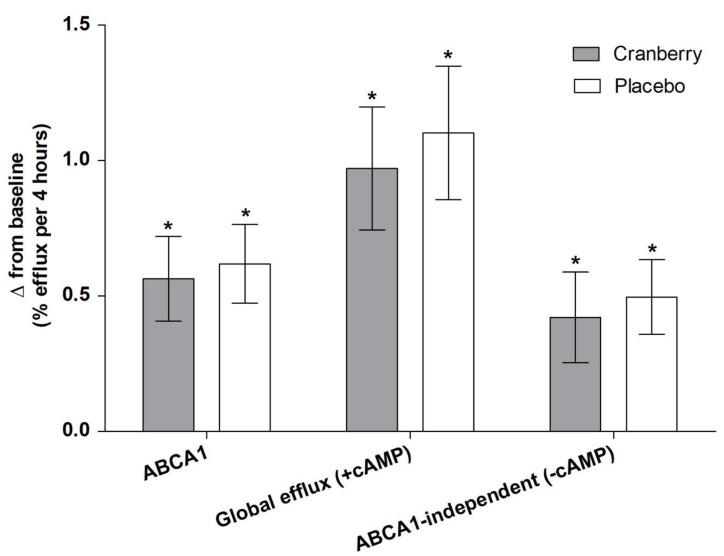
Change in ex vivo measures of HDL-C efflux following supplementation. Change scores were calculated by subtracting study-entry baseline values from each post-supplementation measure. Values are means ± SEM (*n* = 40; 25 M, 15 F) and were compared using the MIXED models procedure (SAS version 9.4; SAS Institute Inc., Cary, NC, USA) to test the effects of treatment, period, and treatment by period interactions. Baseline values were included as covariates for each endpoint. * indicates a significant change from baseline (*p* < 0.05).

There were no significant main effects of treatment on glucose/insulin, lipids, or markers of oxidative stress (Table 3). There was a significant treatment effect for large LDL particles (*p* = 0.02) and LDL size (*p* = 0.001) (Table 4 and Figure 3). Relative to baseline, cranberry juice increased large LDL particles by 8.7% compared to the 2% decrease following the placebo and increased LDL size by 0.3% compared to the 0.3% decrease following the placebo. There was no significant main effect of treatment for any measures of HDL efflux (Table 5 and Figure 4), but all significantly increased from baseline following both cranberry juice and placebo supplementation (*p* ≤ 0.02).

### 3.3. Effects of Cranberry Juice on HDL Efflux and Role of BMI

**Figure 5:**
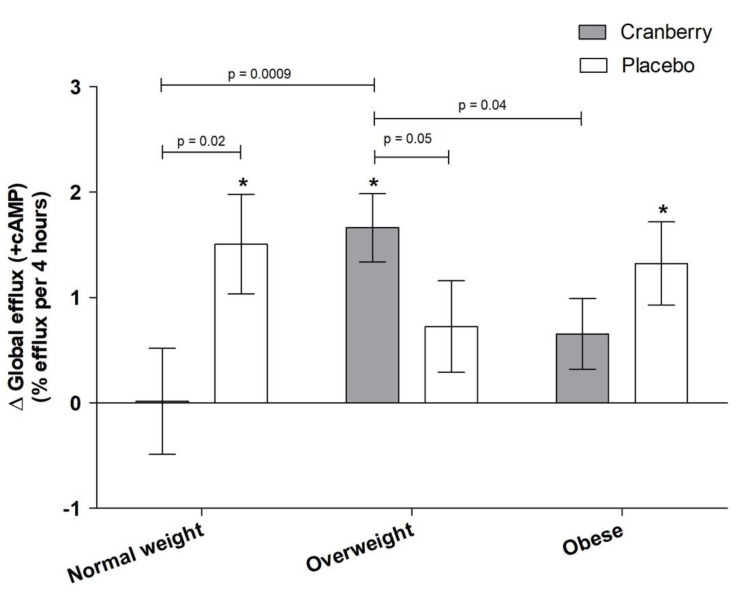
Change in global HDL efflux (+cAMP) following supplementation according to BMI category. Change scores were calculated by subtracting study-entry baseline values from each post-supplementation measure. Values are means ± SEM (*n* = 40; 25 M, 15 F) and were compared using the MIXED models procedure (SAS version 9.4; SAS Institute Inc., Cary, NC, USA) to test the effects of treatment, period, and treatment by period interactions. Baseline values were included as covariates for each endpoint. *p*-values represent unadjusted post hoc comparisons, and * indicates a significant change from baseline (*p* ≤ 0.05).

Exploratory subgroup analyses according to BMI category identified a significant interaction between treatment and BMI for global HDL efflux (+cAMP) (interaction *p*-value = 0.02; Figure 5). Post hoc comparisons demonstrated that cranberry juice supplementation was more effective in participants with overweight compared to both individuals of normal weight and individuals with obesity (*p* = 0.009 and *p* = 0.04, respectively). Cranberry juice also significantly increased global HDL efflux (+cAMP) compared to the placebo in individuals with overweight (*p* = 0.05), whereas there was a greater increase with the placebo in participants of normal body weight (*p* = 0.02). There was no significant difference between the effect of cranberry juice and placebo in individuals with obesity. There was not a significant interaction between treatment and BMI category for other measures of HDL efflux (data not shown; *p* > 0.05).

### 3.4. Baseline CRP as a Moderator of Treatment Effects

**Figure 6:**
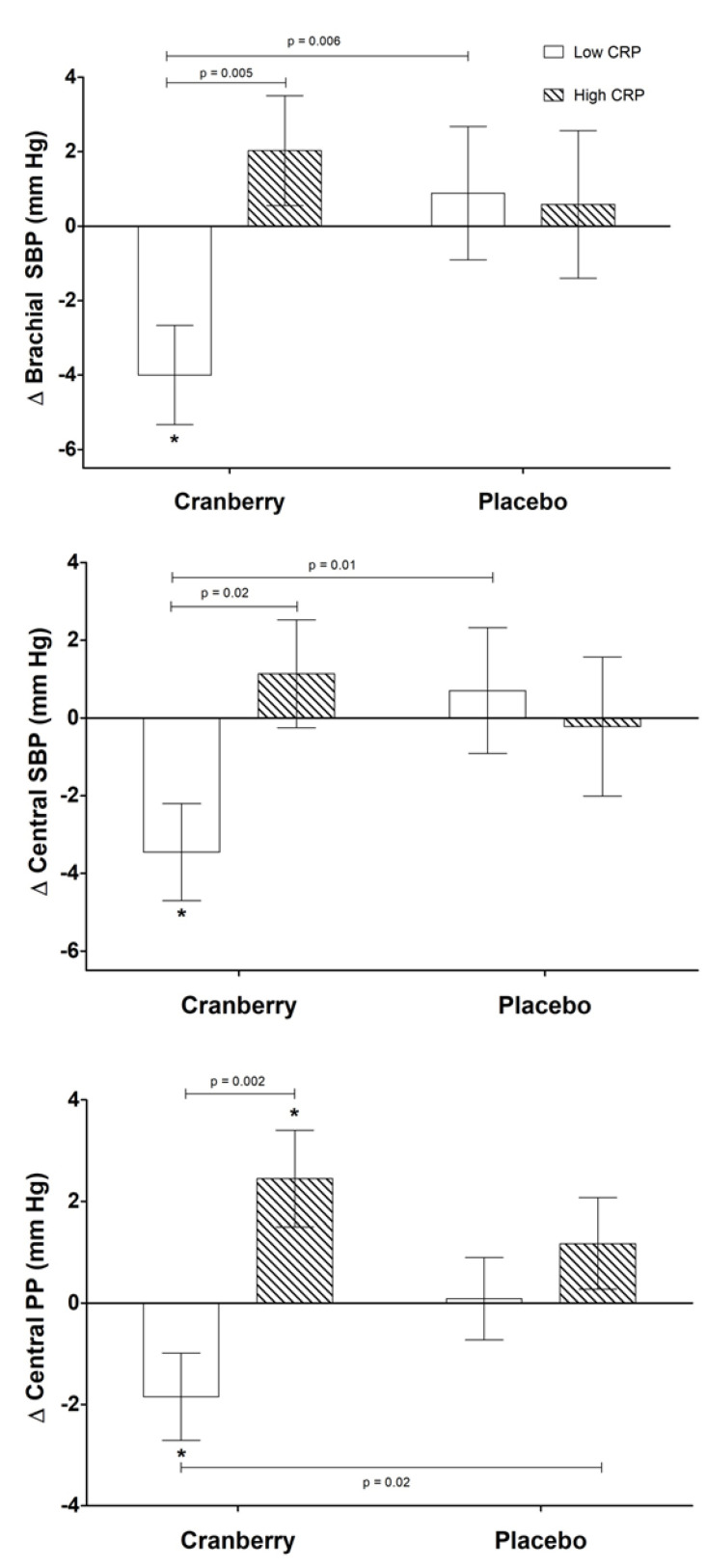
Significant changes in vascular function according to baseline CRP status. Change scores were calculated by subtracting study-entry baseline values from each post-supplementation measure. Values are means ± SEM (*n* = 40; 25 M, 15 F) and were compared using the MIXED models procedure (SAS version 9.4; SAS Institute Inc., Cary, NC, USA). Baseline values were included as covariates for each endpoint. CRP status was defined based on the median baseline CRP value of 1.3 mg/L. *p*-values indicate unadjusted post hoc comparisons, and * indicates a significant change from baseline (*p* ≤ 0.05).

Exploratory secondary analyses revealed significant differences in the effect of supplementation depending on baseline CRP status (defined as high or low compared to the median baseline CRP value of 1.3 mg/L). Baseline characteristics for participants according to CRP status are provided in Table S3. With respect to measures of vascular function, there was a significant treatment by CRP interaction for brachial systolic blood pressure (*p* = 0.001), central systolic blood pressure (*p* = 0.03), and central pulse pressure (*p* = 0.009) (Figure 6). In post hoc analyses, there was a clear distinction in the effect of cranberry supplementation depending on CRP status, with a 4.8% decrease in brachial SBP, 4% decrease in central SBP, and 12% decrease in central PP in individuals with low CRP compared to individuals with high CRP.

There was also a significant treatment by CRP interaction for total cholesterol (*p* = 0.05), HDL-C (*p* = 0.03), non-HDL-C (*p* = 0.04), and glucose (*p* = 0.04), with post hoc analyses indicating clear differences in the response to treatment in individuals with low CRP compared to high CRP (data not shown). Changes in lipoprotein sub-fractions were also influenced by baseline CRP status (data not shown). There was a significant interaction for calculated HDL-C (*p* = 0.007), HDL particles (*p* = 0.01), and the lipoprotein insulin-resistance score (LPIR; *p* = 0.04), a composite metabolic score that incorporates the multi-faceted role of insulin resistance on lipoprotein metabolism [32,33]. In post hoc analyses, calculated HDL-C remained stable in all participants following cranberry supplementation, whereas individuals with high CRP experienced a 4 mg/dL decrease in HDL-C following the placebo compared to individuals with low CRP. In terms of HDL particles, there was a significant decrease following both cranberry and placebo supplementation in individuals with high CRP. For LPIR, cranberry supplementation lowered LPIR by 17% in individuals with low CRP compared to participants with a high baseline CRP.

## 4. Discussion

This study was designed to evaluate the effect of cranberry juice on CVD risk factors in adults with elevated blood pressure. There was no effect of cranberry juice supplementation on the primary endpoint of central systolic blood pressure or for central or brachial diastolic blood pressure. Compared to the placebo, diastolic ambulatory blood pressure was lower following cranberry juice supplementation; the between-treatment difference occurred during daytime hours rather than during nighttime. No other vascular effects were observed. Cranberry juice significantly increased the concentration of large LDL particles and LDL size compared to the placebo. This coincided with a significant decrease from baseline in the concentration of small LDL particles following cranberry juice, but this was not significant compared to the control (treatment *p*-value = 0.09). There was no main effect of treatment on ex vivo HDL efflux, but exploratory subgroup analyses indicate that cranberry juice may be more effective for increasing global HDL efflux (+cAMP) in individuals with overweight compared to those who are normal weight or classified as obese. Our exploratory analyses investigating the effect of baseline CRP also indicate that inflammatory status may potentially alter the effect of cranberry juice supplementation and that there may be greater benefits for vascular function and atherogenic lipoproteins in individuals with lower levels of inflammation.

Previous studies of cranberry supplementation have found mixed results for vascular function endpoints. Epidemiological, in vitro, and animal studies all support the hypothesis that cranberries may be beneficial for vascular function [34,35]—potentially via mechanisms related to their bioactive compounds [5,8]. A recent meta-analysis of cranberry supplementation studies found a significant reduction in brachial systolic blood pressure (−3.6 mm Hg; 95% CI: −6.27, −0.98), an effect that was more apparent in participants who were 50 years or older [8]. Ambulatory blood pressure has been shown to improve CVD risk stratification beyond traditional risk factors, including conventional blood pressure measurement [36,37,38], and previous studies of polyphenol-rich dietary supplements have found significant improvements for both systolic and diastolic ambulatory blood pressure [39,40]. However, null results have been found in other studies [41,42]. To our knowledge, this is the first reported effect for cranberry juice supplementation on ambulatory diastolic blood pressure (as opposed to seated blood pressure). Additional research is needed to verify this effect, but it could present a promising means of CVD risk reduction with cranberry juice consumption.

In terms of central blood pressure and indices of arterial stiffness, clinical studies of antioxidant-rich foods, such as grapefruit juice [43], tart cherry juice [44], blueberries [45,46], pomegranate juice [47,48], and purple potatoes [49], have found significant benefits. Few cranberry studies have reported effects on these endpoints. No effect on AI was found in overweight men with a low-calorie cranberry juice [11], but four weeks of supplementation with double-strength cranberry juice significantly reduced PWV in participants with stable coronary artery disease [12]. Comparatively, our study participants did not have CVD, and the moderately elevated brachial blood pressure of our participants at baseline (mean systolic and diastolic blood pressure = 124/81 mm Hg) may not have been high enough to observe a significant effect of the cranberry treatment [50]. The absence of consistent changes in vascular function and arterial stiffness may also be due to the specific bioactive profile provided by each supplement [39] and/or be related to the plasma bioavailability of cranberry bioactives. Pharmacokinetic studies indicate that plasma availability of cranberry bioactives is very low (<1% of consumed quantities), with high inter-individual variability. Peak plasma concentrations occur over 1–8 h [51,52,53], although there remains debate about whether this occurs earlier (~1–3 h) [53] or later (~6–10 h) [51,52] in that time period. Regardless, this would suggest that the concentration of cranberry bioactives in plasma is very low or non-existent following a 12-h fast and that effects may be more apparent acutely [54]. Furthermore, despite the short half-life of phenolic compounds in plasma, the concentrations are sufficient to induce changes in gene expression and signal transduction [51,53]. Studies have also found that cranberry polyphenol metabolites are abundant in plasma, and these compounds may be the more physiologically active form, although their correlation with vascular function is unclear [52]. There is also evidence of tissue accumulation of anthocyanins in animals following long-term feeding [55], suggesting that repeated exposure over time may confer benefits beyond the acute state. No acute effect of cranberry supplementation has been shown for PWV or AI [52,56], but very few studies have been conducted and in very limited participant populations.

Dietary changes have been shown to be effective for improving the lipid/lipoprotein profile, including LDL-C and HDL-C, but evidence for cranberries remains inconsistent. For instance, a recent meta-analysis of berry-supplementation studies (including nine cranberry studies) found a significant reduction in LDL-C but no change in HDL-C, total cholesterol, triglycerides, apolipoprotein B, or apolipoprotein A-I [57]. Conversely, a meta-analysis focused exclusively on cranberry studies (*n* = 10) found a significant increase in HDL-C, particularly for participants younger than 50 years of age, but no effect on total cholesterol, LDL-C, or triglycerides [8]. With regard to individual studies of cranberry juice, supplementation significantly increased HDL-C in adults with abdominal obesity [13], reduced triglycerides in healthy adults [14], and reduced apolipoprotein B and increased apolipoprotein A-I in patients with type 2 diabetes [15] but had no effect in other studies [16,17,18,19]. Comparatively, we found no effect when using a similar dose of cranberry juice and supplementation duration. It is possible that the baseline lipid profile (mean LDL-C = 124 ± 5 mg/dL) of our study population provided less opportunity to observe significant effects.

Lipoprotein subfractions, defined according to particle size and density, may provide additional insight into CVD risk but do not serve as a replacement for the standard lipid risk factors [58,59,60,61]. With regard to cranberries, supplementation with cranberry juice was found to increase apolipoprotein A-I and decrease apolipoprotein B in patients with type 2 diabetes [15]. We found that cranberry juice significantly increased the concentration of large LDL particles and decreased overall LDL size. This occurred in conjunction with a decrease in small LDL particles compared to baseline, although the main effect of treatment compared to the control did not reach statistical significance (*p* = 0.09). However, given the evidence that LDL size may be a significant factor in assessing CVD risk [58,59], further research into the effects of cranberries on lipoprotein subfractions is warranted.

HDL cholesterol efflux capacity, a measure of reverse cholesterol transport, has been shown to be a predictor of CVD risk and atherosclerosis [20,62,63]. There is some evidence for beneficial effects of cranberries on HDL-C [64,65], but effects on ex vivo HDL efflux have not been reported. Other bioactive-rich supplements have shown mixed results for ex vivo HDL efflux, with significant improvements following anthocyanin supplementation (160 mg/d for 24 weeks) [66] but null results for foods/supplements, such as soy protein [22]. We found no main effect of treatment on either global or transporter-specific cholesterol efflux. However, when participants were stratified by BMI (normal weight, overweight, and obese) in exploratory analyses, there was a significant treatment interaction, with cranberry juice having a greater effect on global efflux in overweight participants compared to those who had obesity or were of normal weight. This could be due to the relative metabolic health of these individuals, with there being no room for improvement in healthy-weight individuals and more severe dysregulation in individuals with obesity that would require more potent interventions. However, this is in contrast to previous studies that have shown more beneficial effects in normal-weight participants [67]. It is also unclear why the placebo juice had a beneficial effect on global cholesterol efflux in the normal-weight participants in the current study compared to cranberry juice. However, this suggests that the baseline health status of participants may play a determining role in the potential effects of cranberry juice supplementation and warrants further investigation.

Similarly, our exploratory analyses suggest that baseline inflammatory status may also determine the effects of cranberry juice supplementation on specified CVD risk factors. Elevated CRP (in the absence of any identifiable acute cause, such as infection, illness, physical trauma, etc.) is indicative of underlying systemic inflammation and higher CVD risk [68]. Thus, individuals with elevated CRP may be experiencing a greater physiological challenge that cannot be overcome by cranberry juice bioactives alone. To our knowledge, no previous studies have investigated a potential interaction with baseline CRP or other inflammatory markers. Additional research is needed to investigate and confirm this potential influence of baseline inflammatory status on the effect of cranberry juice supplementation.

### Strengths and Limitations

The crossover design of this study allowed participants to act as their own controls and minimize the influence of between-subject variability. It should be noted that *p*-values for secondary analyses and post hoc analyses were not corrected for multiple comparisons, which increases the risk of type 1 error. Our sample size was relatively large (*n* = 40), and our participants were recruited specifically on the basis of resting brachial blood pressure to maximize the potential for observing treatment effects relevant to blood pressure management. According to the American Heart Association’s blood pressure categories, the mean brachial blood pressure of our study population (mean = 124/81 mm Hg) would be categorized as hypertensive (i.e., brachial blood pressure 80–89 mm Hg) [69]; however, this degree of blood pressure elevation might have been insufficient to see an effect of the cranberry juice supplement. This and other baseline health characteristics may have reduced the potential for cranberry supplementation to exert significant changes. The cranberry intervention was incorporated into the normal dietary habits of participants, making it more reflective of real world implementation. However, no data were collected on the background diet of participants, and there was no formal oversight to ensure that participants followed the instructions to maintain their normal baseline diet for the duration of the study. This prevents us from conclusively determining whether other diet/lifestyle changes may have occurred and influenced study outcomes. The dose of cranberry juice was consistent with previous studies and represents an easily achievable daily amount. The cranberry and placebo juices were also closely matched for flavor and appearance to maintain blinding and for macronutrients in order to isolate the effect of cranberry bioactives. However, although cranberry juice remains a key source of proanthocyanidins and other bioactive compounds, it should be noted that the multi-step processing methods can lead to a substantial loss of these compounds, particularly anthocyanins, which are more vulnerable to degradation [5,7,70]. Therefore, the type of cranberry supplement (fresh, juice, extract, etc.) may have important implications for clinical effects.

## 5. Conclusions

Supplementation with cranberry juice for eight weeks did not significantly alter the primary endpoint of central systolic blood pressure in adults with overweight and elevated blood pressure; however, the concentration of large LDL particles and LDL size significantly increased without affecting LDL-C concentration. Cranberry juice also significantly lowered 24-h diastolic ambulatory blood pressure. No beneficial effects of cranberry juice were found for central or brachial diastolic pressure or other measures of vascular function. Exploratory subgroup analyses indicated that BMI and CRP may influence effects, and the potential role of baseline health status should be investigated further in future studies. Additional research on other emerging CVD risk factors, such as the gut microbiome, would help to clarify the potential of cranberries for reducing CVD risk.

## Figures and Tables

**Table 1:** Screening characteristics of participants who completed the study (*n* = 40; 25 M, 15 F) and baseline characteristics of participants according to treatment randomization order ^1^.

	Screening Visit	Baseline Visit	
	Pooled	Cranberry-Placebo Sequence	Placebo-Cranberry Sequence
Age (y)	47 ± 12 (30–64)	43 ± 12	50 ± 10
BMI (kg/m^2^)	28.8 ± 4.7 (22.2–39.1)	28.6 ± 4.6	28.9 ± 4.9
*Normal weight (18.5–24.9 kg/m^2^)*	*n* = 9	*n* = 4	*n* = 5
*Overweight (25–29.9 kg/m^2^)*	*n* = 16	*n* = 8	*n* = 8
*Obese (≥30 kg/m^2^)*	*n* = 15	*n* = 6	*n* = 9
Systolic blood pressure (mm Hg)	124 ± 9 (108–139)	126 ± 6	123 ± 13
Diastolic blood pressure (mm Hg)	85 ± 6 (72–97)	82 ± 6	79 ± 8
Glucose (mg/dL)	94 ± 8 (79–113)	91 ± 7	93 ± 7
Total cholesterol (mg/dL)	195 ± 31 (121–268)	184 ± 32	200 ± 42
HDL-C (mg/dL)	49 ± 13 (30–84)	45 ± 11	51 ± 12
TC:HDL-C	4.2 ± 1.2 (1.8–7.8)	4.3 ± 1.0	4.1 ± 1.3
LDL-C (mg/dL)	124 ± 28 (54–176)	115 ± 27	128 ± 39
Triglycerides (mg/dL)	112 ± 46 (51–230)	123 ± 47	99 ± 34

**^1^** Values are means ± SD (*n* = 40; 25 M, 15 F) with ranges in parentheses and were calculated using the univariate procedure (SAS version 9.4; SAS Institute Inc., Cary, NC, USA). There were no significant baseline differences for any values by treatment order (*p* > 0.05). Abbreviations: BMI, body mass index; HDL-C, high-density lipoprotein-cholesterol; LDL-C, low-density lipoprotein-cholesterol; TC, total cholesterol.

**Table 2:** Vascular function and arterial stiffness values in study participants (*n* = 40) following each 8-week supplementation period ^1^.

	Baseline	Cranberry	Placebo	Treatment *p*-Value	Period *p*-Value
Brachial SBP (mm Hg)	124 ± 1	123 ± 1	125 ± 1	0.1	0.2
Brachial DBP (mm Hg)	81 ± 1	79 ± 1	81 ± 1	0.3	0.3
Central SBP (mm Hg)	115 ± 1	113 ± 1	115 ± 1	0.2	0.2
Central DBP (mm Hg)	81 ± 1	80 ± 1	81 ± 1	0.3	0.3
Central PP (mm Hg)	33 ± 1	33 ± 1	34 ± 1	0.5	0.5
Augmentation Pressure (mm Hg)	8.8 ± 0.6	8.5 ± 0.5	8.4 ± 0.4	0.8	0.3
Augmentation Index	21.9 ± 1.5	20.7 ± 1.0	20.7 ± 1.0	>0.9	0.3
Pulse Wave Velocity (m/s)	7.0 ± 0.1	7.1 ± 0.1	7.1 ± 0.1	0.6	0.4
24-h SBP (mm Hg)	124 ± 1	123 ± 1	124 ± 1	0.4	0.3
24-h DBP (mm Hg)	79 ± 1	78 ± 1	80 ± 1	0.05	0.4
24-h PP (mm Hg)	70 ± 1	71 ± 1	70 ± 1	0.3	0.1
Daytime SBP (mm Hg)	129 ± 1	128 ± 1	129 ± 1	0.4	0.2
Daytime DBP (mm Hg)	82 ± 1	81 ± 1	83 ± 1	0.05	0.3
Daytime PP (mm Hg) *	73 ± 1	73 ± 1	72 ± 1	0.3	0.05
Nighttime SBP (mm Hg)	112 ± 2	111 ± 1	109 ± 2	0.4	0.5
Nighttime DBP (mm Hg)	72 ± 1	71 ± 1	71 ± 1	>0.9	0.8
Nighttime PP (mm Hg)	63 ± 1	64 ± 1	64 ± 1	0.7	0.9

^1^ All values are means ± SEM (*n* = 40; 25 M, 15 F) and were compared using the MIXED models procedure (SAS version 9.4; SAS Institute Inc., Cary, NC, USA) to test the effects of treatment, period, and treatment by period interactions. Baseline values were included as covariates for each endpoint. * Period 2 values > Period 1 values. Abbreviations: DBP, diastolic blood pressure; PP, pulse pressure; SBP, systolic blood pressure.

**Table 3:** Blood markers of cardiovascular disease risk in study participants (*n* = 40) following each 8-week supplementation period ^1^.

	Baseline	Cranberry	Placebo	Treatment *p*-Value	Period *p*-Value
Glucose (mg/dL) †	92 ± 1	92 ± 1	94 ± 1 *	0.06	0.04
Insulin	6.4 ± 0.6	6.6 ± 0.4	6.5 ± 0.3	0.9	0.8
Total Cholesterol (mg/dL)	193 ± 6	192 ± 3	192 ± 3	0.8	0.8
HDL-C (mg/dL)	48 ± 2	48 ± 1	48 ± 1	0.8	0.6
TC:HDL-C (mg/dL)	4.2 ± 0.2	4.2 ± 0.1	4.2 ± 0.1	>0.9	0.4
Direct LDL (mg/dL)	124 ± 5	124 ± 2	121 ± 3	0.3	0.7
Non-HDL-C (mg/dL)	144 ± 6	144 ± 2	144 ± 3	0.8	0.6
Triglycerides (mg/dL)	110 ± 7	109 ± 3	115 ± 6	0.3	0.4
CRP (mg/L)	2.3 ± 0.4	2.0 ± 0.3	1.7 ± 0.2	0.3	0.5
Isoprostanes (ng/mL)	0.03 ± 0.001	0.03 ± 0.002	0.023 ± 0.002	0.7	0.5

^1^ All values are means ± SEM (*n* = 40; 25 M, 15 F) and were compared using the MIXED models procedure (SAS version 9.4; SAS Institute Inc., Cary, NC, USA) to test the effects of treatment, period, and treatment by period interactions. Baseline values were included as covariates for each endpoint. * indicates a significant change from baseline (*p* = 0.05). † Period 2 values > Period 1 values. Abbreviations: CRP, C-reactive protein; HDL-C, high-density lipoprotein-cholesterol; LDL-C, low-density lipoprotein-cholesterol; TC, total cholesterol.

**Table 4:** Lipoprotein particle number, particle size, and other lipid profile characteristics in study participants (*n* = 40) following each 8-week supplementation period ^1^.

		Baseline	Cranberry	Placebo	Treatment *p*-Value	Period *p*-Value
VLDL and Chylomicron Particle Concentrations	Total VLDL and Chylomicron Particles (nmol/L)	44.71 ± 1.72	47.40 ± 2.57	50.19 ± 2.29 *	0.3	0.9
	Large VLDL and Chylomicron Particles (nmol/L)	4.42 ± 0.39	4.59 ± 0.35	4.66 ± 0.44	0.9	0.2
	Medium VLDL Particles (nmol/L)	17.63 ± 1.35	17.23 ± 1.48	20.61 ± 1.87	0.06	>0.9
	Small VLDL Particles (nmol/L)	22.68 ± 1.09	25.59 ± 2.00	24.94 ± 1.47	0.7	0.7
LDL Particle Concentrations	Total LDL Particles (nmol/L)	1210.15 ± 38.92	1180.38 ± 22.80	1185.35 ± 30.59	0.9	0.3
	IDL Particles (nmol/L)	245.3 ± 16.73	226.43 ± 16.90	228.65 ± 17.81	0.9	0.5
	Large LDL Particles (nmol/L)	339 ± 18.00	368.53 ± 16.63	332.30 ± 17.61	0.02	0.2
	Small LDL Particles (nmol/L)	625.83 ± 20.13	585.30 ± 20.23 *	624.33 ± 20.51	0.09	0.8
HDL Particle Concentrations	Total HDL Particles (µmol/L)	31.51 ± 0.52	30.87 ± 0.61	31.55 ± 0.52	0.2	0.1
	Large HDL Particles (µmol/L)	6.12 ± 0.32	5.96 ± 0.24	6.00 ± 0.23	0.8	0.9
	Medium HDL Particles (µmol/L)	9.17 ± 0.76	8.84 ± 0.49	8.65 ± 0.69	0.8	0.7
	Small HDL Particles (µmol/L)	16.21 ± 0.70	16.13 ± 0.58	16.84 ± 0.75	0.2	0.03 †
Mean Particle Sizes	VLDL Size (nm)	50.26 ± 0.72	49.84 ± 0.78	49.31 ± 0.89	0.6	0.3
	LDL Size (nm)	20.79 ± 0.06	20.86 ± 0.04	20.72 ± 0.05	0.001	0.7
	HDL Size (nm)	9.15 ± 0.05	9.16 ± 0.04	9.12 ± 0.04	0.3	0.4
Calculated Lipids	Total Triglyceride (mg/dL)	114.6 ± 5.38	115.97 ± 5.13	121.15 ± 6.58	0.4	0.5
	Total VLDL and Chylomicron Triglyceride (mg/dL)	76.7 ± 3.85	78.88 ± 3.79	83.28 ± 4.57	0.4	0.5
	Total HDL Cholesterol (mg/dL)	48.55 ± 1.52	47.83 ± 1.10	47.78 ± 1.1	>0.9	0.3
Lipoprotein Insulin-Resistance Score (LPIR)		50.6 ± 2.28	49.03 ± 1.80	50.10 ± 2.08	0.6	0.2

^1^ All values are means ± SEM (*n* = 40; 25 M, 15 F) and were compared using the MIXED models procedure (SAS version 9.4; SAS Institute Inc., Cary, NC, USA) to test the effects of treatment, period, and treatment by period interactions. Baseline values were included as covariates for each endpoint. * indicates a significant change from baseline (*p* ≤ 0.05). † Period 1 values > Period 2 values. Abbreviations: HDL, high-density lipoprotein; LDL, low-density lipoprotein; VLDL, very low-density lipoprotein, VLDL.

**Table 5:** Ex vivo cholesterol efflux (% efflux per 4 h) in study participants (*n* = 40) following each 8-week supplementation period ^1^.

	Baseline	Cranberry	Placebo	Treatment *p*-Value	Period *p*-Value
ABCA1	2.29 ± 0.12	2.86 ± 0.16 *	2.91 ± 0.15 *	0.8	0.09
Global efflux (+cAMP)	7.19 ± 0.20	8.16 ± 0.23 *	8.29 ± 0.25 *	0.7	0.8
ABCA1-independent (-cAMP)	4.89 ± 0.11	5.30 ± 0.17 *	5.38 ± 0.14 *	0.7	0.2

^1^ All values are means ± SEM (*n* = 40; 25 M, 15 F) and were compared using the MIXED models procedure (SAS version 9.4; SAS Institute Inc., Cary, NC, USA) to test the effects of treatment, period, and treatment by period interactions. Baseline values were included as covariates for each endpoint. ABCA1-specific efflux was calculated as the difference between cAMP-stimulated and non-stimulated (-cAMP) efflux. * indicates a significant change from baseline (*p* ≤ 0.02). Abbreviations: ABCA1, ATP-binding cassette transporter 1; cAMP, cyclic adenosine monophosphate.

## Data Availability

The data presented in this study are available on request from the corresponding author.

## Supplementary Materials

The following are available online at https://www.mdpi.com/article/10.3390/nu13082618/s1, Table S1: Nutrient profile of the cranberry and placebo beverages, Table S2: Phenolic content of cranberry and placebo beverages (mg per 8 fl oz), Table S3: Baseline characteristics of study participants by CRP status, Figure S1: Significant treatment by sex interactions following supplementation.
