# Supplementary material for: Effects of Cranberry Juice Supplementation on Cardiovascular Disease Risk Factors in Adults with Elevated Blood Pressure: A Randomized Controlled Trial
Source: Nutrients. 2021 Jul 29;13(8):2618. doi: 10.3390/nu13082618 (PMC8398037; doi:10.3390/nu13082618)

Supplemental Table S1. Nutrient profile of the cranberry and placebo beverages. [1]

	**Cranberry Juice[2]**	**Placebo Juice[3]**
**Calories (kcal)**	34.73	33.79
**Protein (g)**	0.00	0.00
**Carbohydrate (g)**	8.76	8.40
**Fat (g)**	0.00	0.00
**SFA (g)**	0.00	0.00
**Trans fat (g)**	0.00	0.00
**Cholesterol (mg)**	0.00	0.00
**Pro-Vitamin A (IU)**	0.91	0.00
**Vitamin C (mg)**	0.00	0.00
**Vitamin D (IU)**	0.00	0.00
**Calcium (mg)**	10.61	1.97
**Sodium (mg)**	47.18	42.70
**Potassium (mg)**	51.53	64.10

[1]per 8 fl oz serving

[2]Contained 27% cranberry juice content, fructose, pectin, citrate salts, and flavoring. Total sugar: 6.26g; Added sugar: 4.57 g

[3]Contained water, fructose, dextrose, citric acid, malic acid, pectin, citrate salts, flavoring, and colorants to match the appearance, aroma and taste of the cranberry beverage in the absence of cranberry juice. Total sugar: 7.41 g; Added sugar: 5.58 g

Supplemental Table S2. Phenolic content of cranberry and placebo beverages (mg per 8 fl oz).

	**Cranberry Juice**	**Placebo Juice**
Total anthocyanins	2.27	ND
Total phenolic acids	33.77	ND
Total flavonols	8.46	ND
*PACs (OSC-DMAC method)	130.17	ND
**PACs (BL-DMAC method)	39.54	ND
Total phenolics	159.62	ND

PACs, proanthocyanidins; ND, non-detectable
*with PACs extracted from Cranberry juice as the standard
**with PAC A2 dimer as standard

Supplemental Table S3. Baseline characteristics of study participants by CRP status.[1]

	**Low CRP (≤1.3 mg/L; n = 22)**	**High CRP (>1.3 mg/L; n = 18)**
**Age (yrs)**	48 ± 11 (30 – 64)	46 ± 12 (30 – 64)
**BMI (kg/m^2)**	27.3 ± 4.1 (22.2 – 35.6)	30.6 ± 4.8 (23.2 – 39.1)
Normal weight (18.5 – 24.9 kg/m^2)	n = 7	n = 3
Overweight (25 – 29.9 kg/m^2)	n = 10	n = 5
Obese (≥30 kg/m^2)	n = 5	n = 10
**Systolic blood pressure (mm Hg)**	122 ± 10 (103 – 140)	127 ± 10 (112 – 150)
**Diastolic blood pressure (mm Hg)**	91 ± 6 (81 – 90)	83 ± 8 (71 – 97)
**Glucose (mg/dL)**	94 ± 8 (79 – 103)	93 ± 8 (80 – 110)
**Insulin (IU)**	5.2 ± 3.0 (1.3 – 12.0)	8.0 ± 3.9 (2.2 – 17.2)
**Total cholesterol (mg/dL)**	189 ± 40 (133 – 293)	197 ± 37 (113 – 255)
**HDL-C (mg/dL)**	48 ± 14 (29 – 80)	48 ± 10 (38 – 73)
**Non-HDL-C (mg/dL)**	141 ± 39 (69 – 238)	149 ± 36 (72 – 213)
**TC:HDL**	4.2 ± 1.3 (1.9 – 7.6)	4.2 ± 1.1 (2.7 – 6.2)
**LDL-C (mg/dL)**	120 ± 36 (56 – 219)	126 ± 34 (57 – 189)
**Triglycerides (mg/dL)**	105 ± 41 (51 – 202)	116 ± 43 (66 – 206)

[1]Values represent mean ± SD with ranges in parentheses. CRP status was defined based on the median baseline CRP value of 1.3 mg/L.

Supplemental Figure S1. Significant treatment by sex interactions following supplementation. P-values represent unadjusted *post hoc* comparisons and * indicates a significant change from baseline ($p \leq 0.05$).

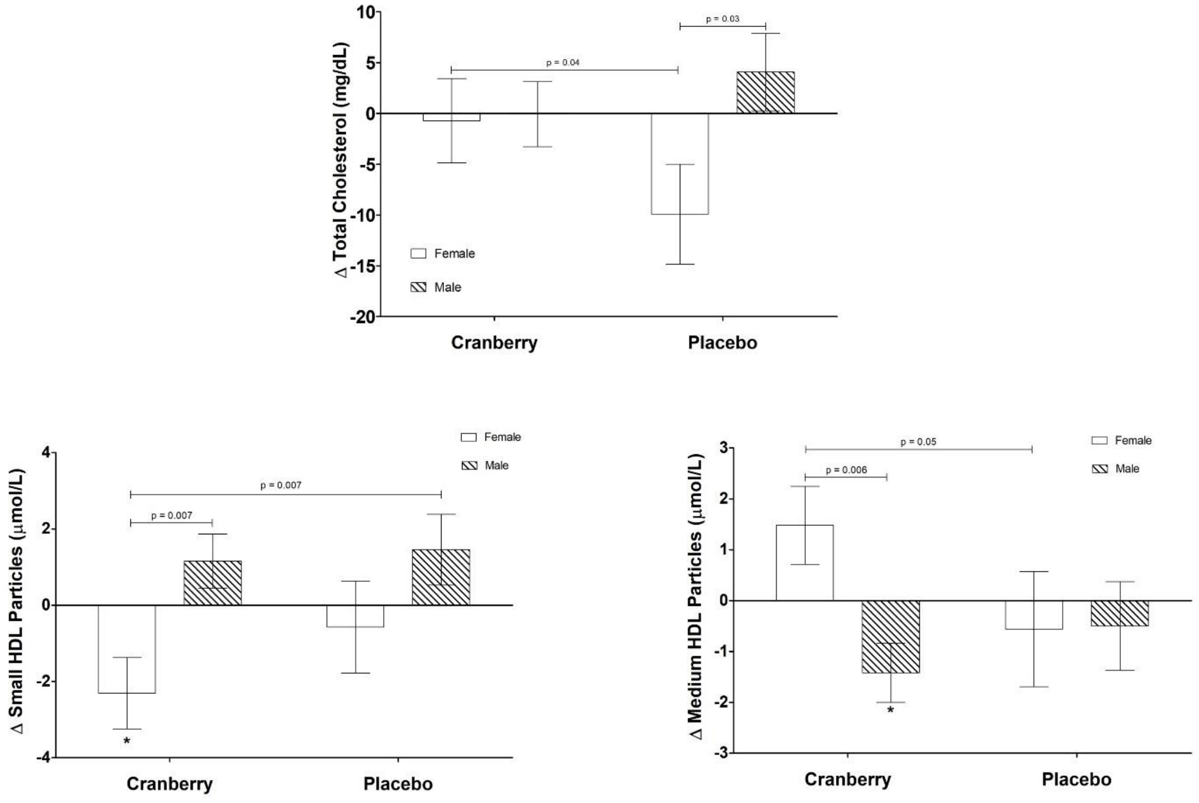

Supplement: Supplementary file 1 [file nutrients-13-02618-s001.zip › nutrients-1280280-supplementary.pdf]
